# Phosphorylation of AKT by lysyl oxidase-like 2 activates the PI3K/AKT signaling pathway to promote proliferation, invasion and metastasis in esophageal squamous carcinoma

**DOI:** 10.1007/s12094-023-03133-5

**Published:** 2023-03-30

**Authors:** Zhiqin Fan, Yingmin Liu, Xinya Liu, Wei Nian, Xiaotong Huang, Qianqian Yang, Songyu Hou, Fei Chen

**Affiliations:** 1 Department of Daily Surgery, Affiliated Tumor Hospital, Xinjiang Medical University, Urumqi, China; 2 Department of Cardiac Oncology Disease, Affiliated Tumor Hospital, Xinjiang Medical University, Urumqi, China; 3 State Key Laboratory of Pathogenesis, Prevention and Treatment of High Incidence Diseases in Central Asia, Xinjiang Medical University, Urumqi, China

**Keywords:** ESCC, LOXL2, Phosphorylation of AKT, PI3K/AKT signaling pathway, Proliferation, Invasion, Metastasis

## Abstract

**Objective:**

Esophageal squamous cell carcinoma (ESCC) is a common and aggressive malignancy of the gastrointestinal tract for which therapeutic options are scarce. This study screens for LOXL2, a key gene in ESCC, and explains the molecular mechanism by which it promotes the progression of ESCC.

**Methods:**

Immunohistochemical staining was performed to detect the expression level of LOXL2 in ESCC tissues and paraneoplastic tissues. CCK-8 and Transwell assays were performed to assess the effects of LOXL2 knockdown and overexpression on the proliferation, apoptosis, migration and invasion ability of ESCC cells. High-throughput sequencing analysis screens for molecular mechanisms of action by which LOXL2 promotes ESCC progression. Western blotting and qRT-PCR were used to determine the expression levels of relevant markers.

**Results:**

LOXL2 is positively expressed in ESCC and highly correlated with poor prognosis. Silencing LOXL2 significantly inhibited the proliferation, migration and invasive ability of ESCC cells, whereas overexpression showed the opposite phenotype. High-throughput sequencing suggested that LOXL2-associated differentially expressed genes were highly enriched in the PI3K/AKT signaling pathway. In vitro cellular assays confirmed that silencing LOXL2 significantly reduced PI3K, p-AKT^Thr308^ and p-AKT^Ser473^ gene and protein expression levels, while overexpression increased all three gene and protein levels, while AKT gene and protein expression levels were not significantly different.

**Conclusion:**

This study found that LOXL2 may regulate the PI3K/AKT signaling pathway and exert protumor effects on ESCC cells through phosphorylation of AKT. LOXL2 may be a key clinical warning biomarker or therapeutic target for ESCC.

## Introduction

Esophageal squamous carcinoma (ESCC) is one of the most common malignancies of the upper gastrointestinal system and the leading cause of cancer-related deaths worldwide [1, 2]. Early clinical symptoms of esophageal squamous carcinoma are not obvious, and 70% of patients have already developed local infiltration or distant metastases by the time they are seen [3–5]. Although the application of new technologies and neoadjuvant therapy has improved the survival rate of ESCC patients, the prognosis of ESCC remains poor, with a 5-year survival rate of approximately 20% [6–8]. Current studies lack clinical early warning diagnostic biomarkers for ESCC; therefore, it is important to establish effective early warning biomarkers.

Lysyl oxidase-like 2 (LOXL2) is a member of the lysyl oxidase (LOX) family [9], which functions to catalyze the cross-linking of elastin and collagen in the extracellular matrix (ECM) and has other intracellular functions associated with fibrogenic and carcinogenic effects [10, 11]. Related studies have shown that LOXL2 expression is significantly altered in breast cancer [12], aggressive osteosarcoma [13], lung cancer [14], pancreatic ductal adenocarcinoma [15], colorectal cancer [16], and prostate cancer [17], and has a significant effect on the malignant biological behavior of tumors. Although the biological functions of LOXL2 have been identified in a variety of cancer types, the molecular mechanisms of LOXL2 in ESCC are unknown.

The phosphatidylinositol 3-kinase/protein kinase-B (PI3K/AKT) pathway is an important intracellular signaling pathway [18]. This process is mediated by serine or threonine phosphorylation of a range of downstream substrates, and the key genes involved are PI3K and AKT, so this pathway is directly named after these two genes [19]. The main role of the PI3K/AKT signaling pathway is to promote metabolism, proliferation, cell survival, growth and angiogenesis in response to extracellular signals [20, 21]. PIP3 activates serine/threonine kinase, phosphoinositide-dependent protein kinase-1 (PDK1) and Akt. Activated PIP3 recruits cytoplasmic serine/threonine kinase and transports it to the cell membrane by binding to the N-terminal PH domain of AKT. PI3K works with PDK1 to phosphorylate the serine phosphorylation site (Ser473) and the threonine phosphorylation site (Thr308) of AKT to activate AKT. Activated AKT enters the nucleus and activates or inhibits a variety of downstream proteins involved in the regulation of cell survival, proliferation, apoptosis and angiogenesis [22–25]. It has been shown that the PI3K/AKT signaling pathway is dysregulated in almost all human cancers, such as breast cancer [26], gastrointestinal cancer [27], colorectal cancer [28] and ovarian cancer [29], which highlights the potential therapeutic value of this pathway in cancer therapy [30].

In this study, we analyzed the clinical significance of LOXL2 in esophageal squamous carcinoma through clinicopathological data, applied high-throughput sequencing to screen LOXL2 and the PI3K/AKT pathway, and explored the effect of altered LOXL2 gene expression on the biological characteristics of ESCC cells through in vitro cell experiments. The effect of altered LOXL2 gene expression on the biological properties of ESCC cells was investigated through ex vivo cellular assays, and the mechanism by which LOXL2 activates the PI3K/AKT signaling pathway through phosphorylation of AKT to promote the proliferation and metastasis of esophageal squamous carcinoma was elucidated.

## Material and method reagents

### Collection of esophageal squamous carcinoma specimens

A total of 37 pairs of human esophageal squamous carcinoma and matched paracancerous tissue specimens were collected from the Affiliated Tumor Hospital, Xinjiang Medical University, (Xinjiang Uygur Autonomous Region, China) following radical esophageal cancer surgery. None of the patients were treated with radiotherapy before surgery. Informed consent was obtained for the collection of specimens, which was approved by the Institutional Review Board. The experiments were conducted in accordance with the ethical standards of the 1964 Declaration of Helsinki and its subsequent amendments or similar ethical standards.

### Materials

KYSE450, KYSE510, KYSE30, and TE-1 human esophageal cancer cells were purchased from Shanghai YaJi Biological. The human esophageal carcinoma cell lines ECA109 and KYSE150 were purchased from Pricella Biological. Fetal bovine serum (FBS) was purchased from Excell Bio. DMEM (high sugar) medium and trypsin (0.25% Trypsin–EDTA) were purchased from GIBCO Biological. A CCK-8 cell proliferation/virulence assay kit was purchased from TransGen Biotech. The viral coinfection reagent HitransG A and viral coinfection reagent HitransG P were purchased from GeneChem Biological. Transwells were purchased from Corning Biological. Anti-LOXL2 antibody was purchased from Boster Biological. Anti-PI 3-kinase p85 alpha antibody, anti-AKT1 antibody, anti-phospho-AKT1 (Ser473), and anti-phospho-Akt (Thr308) antibody were purchased from Bioss Biological (Table 1).

**Table 1:** Experimental reagents and consumables

Reagents/consumables	Manufacturers	Goods number
KYSE450	Shanghai YaJi	YS3155C
KYSE510	Shanghai YaJi	YS2921C
KYSE30	Shanghai YaJi	YS321C
TE-1	Shanghai YaJi	YS290C
ECA109	Pricella	CL-0077
KYSE150	Pricella	CL-0638
Fetal bovine serum (FBS)	Excell Bio	FND500
DMEM (high sugar) medium	GIBCO	C11995500BT
Trypsin (0.25% Trypsin–EDTA)	GIBCO	25200-056
CCK-8 cell proliferation/virulence assay kit	TransGen	FC101-03
Viral coinfection reagent HitransG A	Genechem	REVG004
Viral coinfection reagent HitransG P	Genechem	REVG005
Transwells	Corning	3422
Anti-LOXL2 antibody	Boster	PB0759
Anti-PI 3 Kinase p85 alpha antibody	Bioss	bs-0128R
Anti-AKT1 antibody	Bioss	bs-0115R
Anti-phospho-Akt (Ser473) antibody	Bioss	bs-12456R
Anti-phospho-Akt (Thr308) antibody	Bioss	bs-2720R

### High-throughput sequencing

One milliliter of TRIzol reagent was added to each vial of cells to extract total RNA from the cells. RNA integrity was accurately detected using an Agilent 2100 RNA Nano 6000 Assay Kit (Agilent Technologies, CA, USA). Three micrograms of total RNA per sample was used as the starting material for library construction, and upon completion, initial quantification was performed using Qubit 3.0. Agilent 2100 for detection. Bio-Rad KIT iQ SYBR GRN was used for Q-PCR. The effective library concentration was accurately quantified (effective library concentration > 10 nM). The different libraries were pooled into a flow cell according to the effective concentration and the target downstream data volume needed. cBOT was clustered and sequenced using the Illumina high-throughput sequencing platform (HiSeq/MiSeq).

### Immunohistochemistry

LOXL2 protein expression levels were measured in esophageal squamous carcinoma specimens and paraneoplastic specimens by immunohistochemistry. LOXL2 antibody was purchased from ORIGENE, USA (No. TA807443). The results were interpreted in a double-blind manner by two senior pathologists, and the results were averaged. LOXL2 was positive for the presence of brownish-yellow granules in the cytoplasm and was classified into 4 grades according to the proportion of positive cells and the degree of staining: (1) cancer cells with no cell membrane/cytoplasm/nucleus staining or ≤ 10% of cancer cells showing a (−) cell membrane/cytoplasm/nucleus staining. (2) (+) for > 10% of cancer cells showing partial staining of cytoplasm. (3) > 10% of cancer cells showing weak or moderate complete staining of cytoplasm as (+ +). (4) > 10% of the cancer cells show highly complete staining of the cytoplasm (+ + + +). When (−) was judged as the non-expression group that was negative, (+) to (+ + + +) was judged as the high expression group that was positive.

### Lentiviral transfection

Two milliliters of cell suspension at a density of 5 × 10^4^ cells/mL was prepared in complete medium. Then, 100 μL/well was added to a 96-well plate, 12 wells in total, 3 of which were used as the control group, and incubated at 37 °C for 24 h until the cell confluence was approximately 30%. The virus was diluted to a titer of 1 × 10^8^ TU/mL, 1 × 10^7^ TU/mL and 1 × 10^6^ TU/mL with serum-free medium, 50 μL each. Aspirate the supernatant from each well, add the virus and the corresponding infection-enhancing solution, mix well, and continue to incubate. After 12 h of infection, the culture medium was returned to normal. After approximately 48 h of infection, when the fluorescence expression was high, the wells were observed by microscopy. The infection conditions and MOI corresponding to the group with an infection efficiency of approximately 80% and good cell growth can be used as the basis for subsequent infection experiments.

### CCK-8 proliferation assay

TE-1 and KYSE30 cells in good growth conditions with 90% confluence were digested with trypsin. Complete medium was prepared into a single-cell suspension of 5 × 10^4^ cells/mL, and the cells were inoculated into 96-well plates (100 μL/well). The cells were incubated for 24 h at 37 °C in 5% CO2, the medium was discarded, and the interventions were carried out in groups of 5 replicates each. After the intervention, the medium was discarded, and 100 μL of 10% CCK-8 solution was added to each well. The incubation was continued in the incubator, and the OD value at 450 nm was measured after 1 h using an enzyme marker.

### Cell-cycle assay

Interventions were performed according to the experimental groups. The cells were collected, washed once in PBS, and resuspended in 500 μL of prechilled PBS. The cell suspension was added to 3.5 ml of precooled 80% ethanol and fixed overnight at 4 °C. The samples were centrifuged at 2000 rpm for 5 min, and the supernatant was carefully aspirated. The cells were washed 2 times with prechilled PBS, and the supernatant was discarded. The cells were resuspended by adding 500 μL PI/RNase Staining Buffer. The samples were screened with a 200 mesh nylon sieve, and a single-cell suspension was made. The samples were incubated for 30 min at 4 °C in the dark. Flow cytometry was used to detect red fluorescence at an excitation wavelength of 488 nm and to detect light scattering. Analysis software was used for cellular DNA content analysis.

### Apoptosis detection

The interventions were carried out according to the experimental groups. The culture fluid from each group of cell bottles was aspirated into a centrifuge tube (containing apoptotic or necrotic cells that were suspended). PBS was used to wash the walled cells twice. The PBS was collected together in a centrifuge tube, and the cells were digested with trypsin. The cells were transferred to a centrifuge tube and centrifuged at 1000 rpm for 5 min, and the supernatant was discarded. The cells were washed twice with precooled PBS, and the clean supernatant was discarded. Then, 500 μL of 1 × binding buffer was added to resuspend the cells, and the cells were passed through a 200 mesh sieve to make a single-cell suspension. Then, 5 μL of Annexin V-PE and 10 μL of 7-AAD were added to each tube and mixed gently. The plates were incubated at 4 °C for 10 min away from light. Flow cytometry assays were performed within 30 min.

### Cell migration assay

Interventions were performed, and cells were collected according to experimental groupings. The cells were washed once with PBS and serum-free medium successively. The cells were resuspended in serum-free medium, and the cell suspension was adjusted to the appropriate density. Add 600 μl of complete medium to the lower chamber (i.e., the bottom of the 24-well plate) and 100 μl of cell suspension to the upper chamber. Three replicate wells per group were incubated in the incubator for the time determined by the results of the pretest. The chambers were carefully removed with forceps and washed 2 times with PBS. The cells were fixed in 4% formaldehyde for 20 min at room temperature. The chambers were removed and washed 2 times with PBS. Transfer to wells prefilled with 400 μl of Giemsa staining solution A. Staining was performed for 1 min at room temperature. Add 800 μl of Giemsa stain B and continue staining for 5 min. The chamber was removed and washed twice with PBS. The cells were carefully wiped off from the surface of the upper chamber with a damp cotton swab and dried with the bottom side up. Cells were transferred to a slide, and cell migration was observed under a microscope.

### Cell invasion assay

The matrix gel was melted overnight at 4 °C in advance. The matrix gel was diluted with precooled serum-free medium at 4 °C to a final concentration of 1 mg/ml. Add 100 μl of the diluted matrix gel vertically to the bottom center of the upper chamber of the chamber. The samples were incubated at 37 °C for 5 h to dry to a gelatinous consistency. Interventions were carried out, and cells were collected according to experimental groupings and successively washed once with PBS and serum-free medium. The cell suspension was adjusted to the appropriate density by suspending the cells in serum-free medium. Add 600 μl of complete medium to the lower chamber (i.e., the bottom of the 24-well plate) and 100 μl of cell suspension to the upper chamber.

Add 100 μl of cell suspension to the upper chamber. Three replicate wells per group were incubated in the incubator for the time determined by the results of the pretest. The chambers were carefully removed with forceps, washed twice with PBS and fixed for 20 min at room temperature in 4% formaldehyde. The chambers were removed, washed twice with PBS, transferred to wells prefilled with 400 μl of Giemsa stain A solution and stained for 1 min at room temperature. Then, 800 μl of Giemsa stain B was added, and staining was continued for 5 min. The chamber was removed and washed 2 times with PBS. The cells were carefully wiped off from the surface of the upper chamber with a damp cotton swab and dried with the bottom side up. The cells were transferred to a slide, and cell invasion was observed under a microscope.

### Statistical treatment

SPSS 25.0 software was used to analyze the experimental results. Data are expressed as the mean ± standard deviation. One-way ANOVA was used to evaluate the differences between experimental groups. *P* < 0.05 was considered a statistically significant difference.

## Results

### Positive expression of LOXL2 in ESCC tissue is positively associated with poor prognosis

**Fig. 1:**
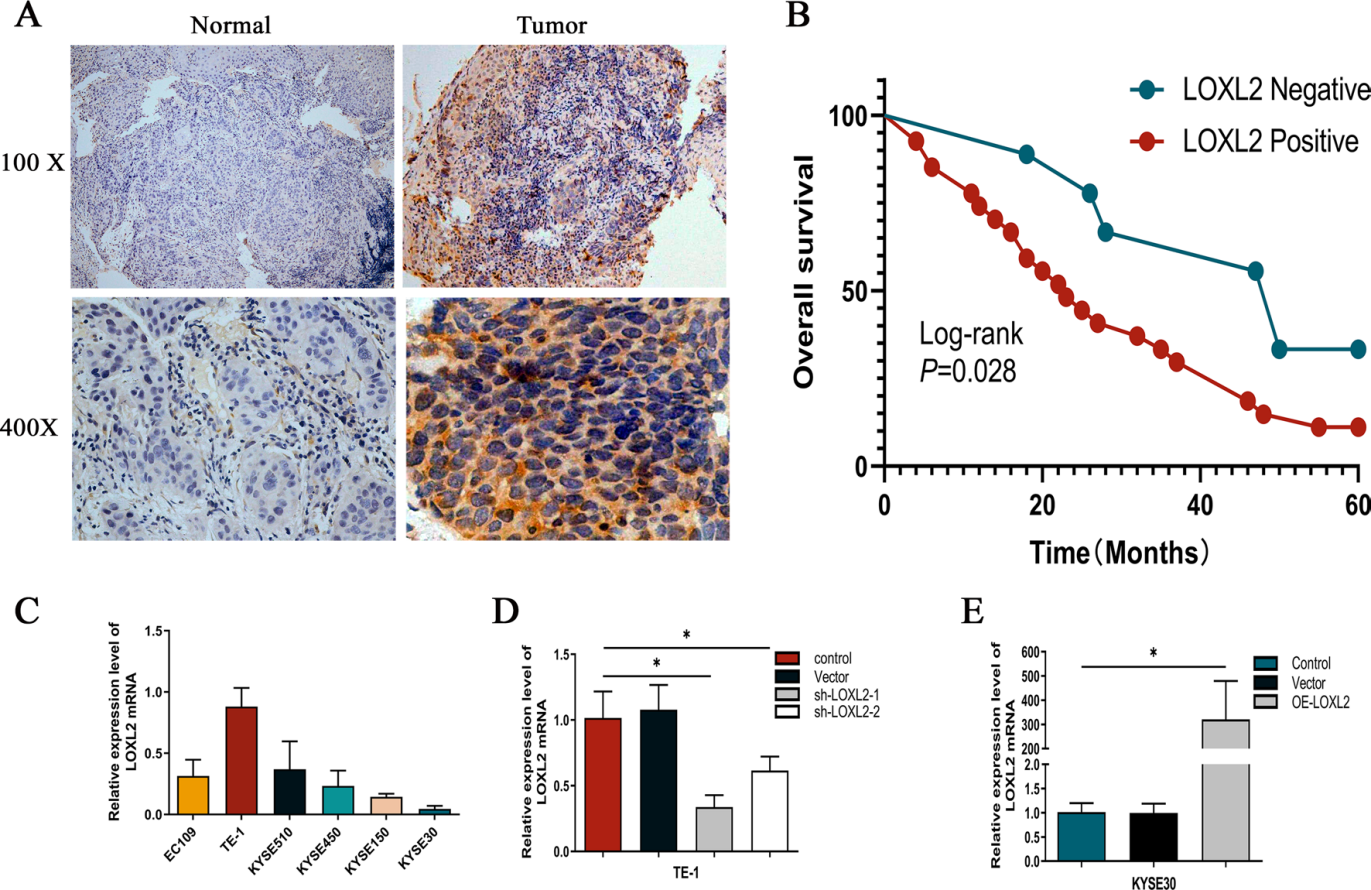
LOXL2 was positively expressed in ESCC tissues and positively correlated with poor prognosis. **a** LOXL2 was significantly more highly expressed in ESCC tissues than in paired paracancerous tissues. **b** Kaplan‒Meier survival curves of ESCC patients correlated with LOXL2 expression. **c** RT-qPCR was performed to detect LOXL2 expression levels in ESCC cell lines. **d** TE-1 cells were transfected with sh-LOXL2 lentivirus, and LOXL2 expression was measured. **e** KYSE30 cells were transfected with LV-LOXL2 lentivirus, and LOXL2 expression was measured

To explore the phenotypic expression and clinical significance of LOXL2 in ESCC progression, this study applied immunohistochemical techniques to detect LOXL2 expression levels in ESCC and paracancerous specimens from 37 patients with stage III disease, and the results showed that LOXL2 was significantly upregulated in esophageal squamous carcinoma tissues (Fig. 1a). Meanwhile, Kaplan‒Meier curve analysis was performed on clinical prognostic data, and the results suggested that the overall survival rate of patients in the LOXL2-positive expression group was significantly lower than that of the control group, which was highly correlated with poor prognosis (Fig. 1b). The correlation between LOXL2-positive expression and clinicopathological characteristics of patients was analyzed as shown in Table 2, and LOXL2-positive expression was associated with ESCC T stage (*p* = 0.003) and lymphatic metastasis (*p* = 0.010), but no differences were observed in patient age, sex, CEA expression status or tumor. In conclusion, this study suggests that LOXL2 plays an important role in the progression of ESCC. To further analyze the effect of LOXL2 on ESCC cell function, we first assessed the LOXL2 mRNA expression levels in six ESCC cell lines (ECA109, TE-1, KYSE510, KYSE450, KYSE150 and KYSE30). (Fig. 1c) and was, therefore, selected for further analysis. To examine the function of LOXL2, LOXL2-shRNA (80,056–1) and LOXL2-shRNA (80057-1) lentiviruses were used to infect TE-1 cells, LV-LOXL2 lentiviruses were used to transfect KYSE30 cells, and the efficiency of LOXL2 knockdown and overexpression was confirmed by RT‒qPCR. The final stable LOXL2 knockdown and overexpression cell lines were constructed (Fig. 1d, e).

**Table 2:** Relationship between LOXL2 expression status and clinicopathological features in patients with esophageal squamous carcinoma

Characteristics	All case (*n* = 37)	LOXL2 expression status		*χ*^*2*^	*P* values
		Negative (*n* = 9)	Positive (*n* = 28)		
Sex					
Male	26	4	22	3.797	0.051
Female	11	5	6		
Age/years					
< 60	15	3	12	0.256	0.613
≥ 60	22	6	16		
CEA					
Negative	36	9	27	0.330	0.565
Positive	1	0	1		
Histological grade					
Well	2	0	2	1.170	0.557
Moderate	19	4	15		
Poor	16	5	11		
T stage					
T2	11	6	5	11.566	0.003
T3	22	1	21		
T4	4	2	2		
Lymphatic metastasis					
Negative	2	2	0	6.578	0.010
Positive	35	7	28		

*CEA* carcinoembryonic antigen

### LOXL2 upregulates the proliferative capacity of ESCC cells and participates in cell cycle differentiation

**Fig. 2:**
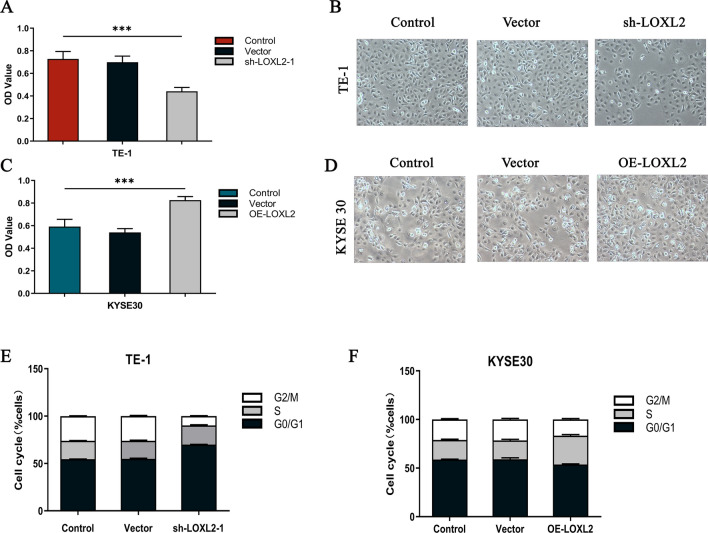
LOXL2 upregulates the proliferative capacity of ESCC cells and their involvement in cell cycle differentiation. **a**, **b** The proliferative capacity of TE-1 cells was analyzed by Cell Counting Kit-8 assay, and the proliferative capacity of ESCC cells was significantly reduced after silencing LOXL2. **c**, **d** The proliferation ability of KYSE30 cells was analyzed by Cell Counting Kit-8 assay, and the proliferation ability of ESCC cells was significantly enhanced after LOXL2 overexpression. **e**, **f** TE-1 and KYSE30 cells were analyzed by flow cytometry to determine the percentage of cells in each phase of the cell cycle. P values were determined by Pearson correlation, *P < 0.05, **P < 0.01, ***P < 0.001

Clinical data showed that positive LOXL2 expression was associated with tumor proliferation, so the effect of LOXL2 silencing and overexpression on ESCC cell proliferation was examined using the Cell Counting Kit-8 (CCK-8) assay. The results showed that silencing LOXL2 in TE-1 cells significantly reduced ESCC cell growth in vitro (*P* < 0.001) (Fig. 2a, b), while overexpression of LOXL2 in KYSE30 cells significantly enhanced ESCC cell growth in vitro (P < 0.001) (Fig. 2c, d). To investigate the mechanism by which LOXL2 affects the proliferation of ESCC cells, the cell cycle distribution was analyzed using flow cytometry. Silencing LOXL2 in TE-11 cells led to an increase in G0/G1 phase cells and a decrease in the proportion of S phase cells compared to control cells (Fig. 2e), while overexpression of LOXL2 in KYSE30 cells led to a decrease in G0/G1 phase cells and an increase in the proportion of S phase cells compared to control cells (Fig. 2f). These results suggest that LOXL2 upregulates the proliferative capacity of ESCC and participates in the cell cycle differentiation process.

### LOXL2 promotes apoptosis, migration and invasion of ESCC cells

**Fig. 3:**
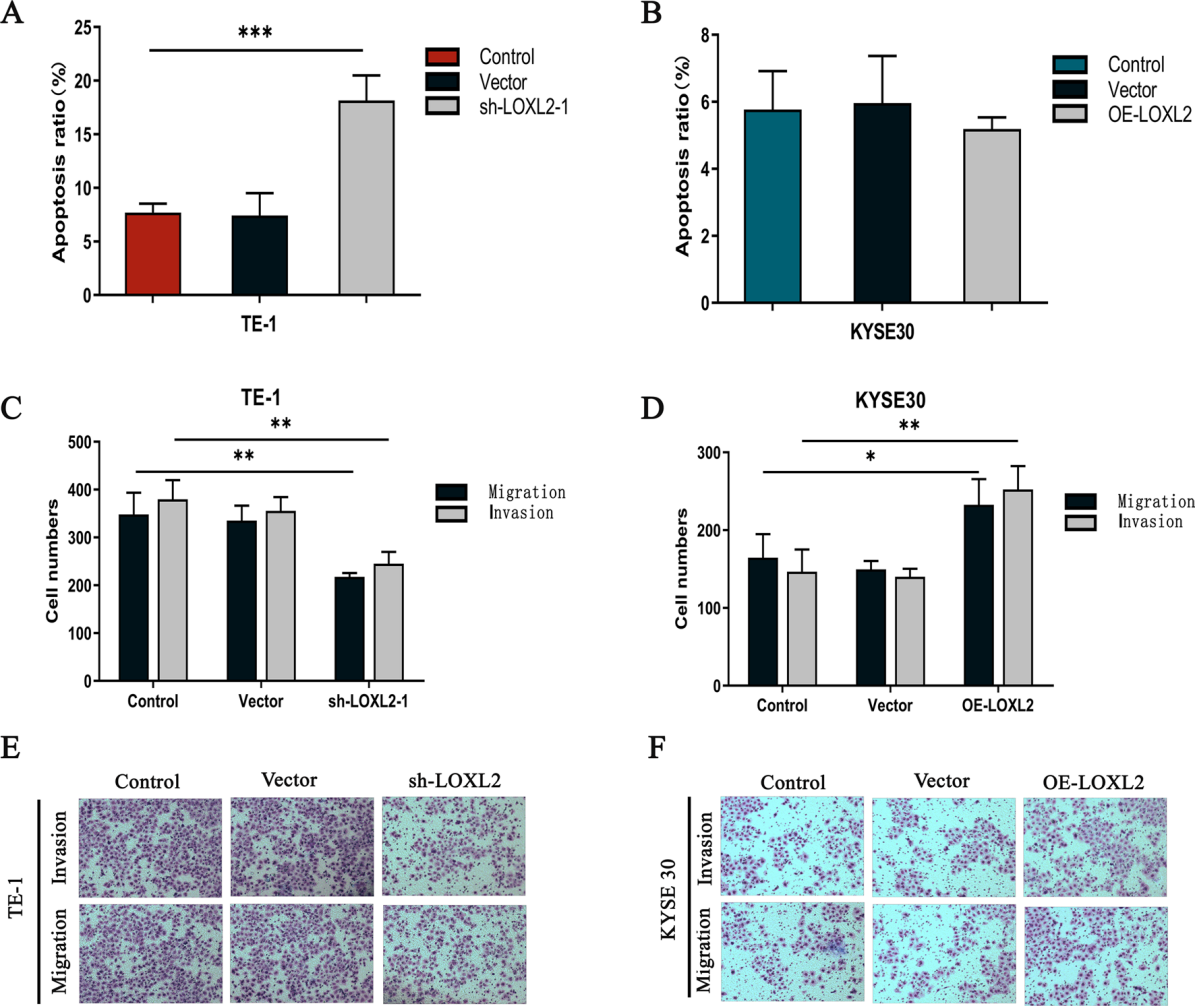
LOXL2 is involved in the regulation of apoptosis, migration and invasion of ESCC cells. **a** Transwell assays were used to analyze the apoptotic ability of TE-1 cells, and the apoptotic ability of ESCC cells was significantly enhanced after LOXL2 knockdown. **b** Transwell assays were performed to analyze the apoptotic ability of KYSE30 cells, and there was no significant change in apoptotic ability after LOXL2 overexpression. **c**, **e** Transwell assays were used to analyze the migration and invasion abilities of TE-1 cells, and the migration and invasion abilities of ESCC cells were significantly reduced after silencing LOXL2. **d**, **f** Transwell assays were used to analyze the migration and invasion abilities of KYSE30 cells, and the migration and invasion abilities of ESCC cells were enhanced after LOXL2 overexpression. P values were determined by Pearson correlation, *P < 0.05, **P < *0.01*, ***P < 0.001

Clinical data suggested that positive LOXL2 expression was associated with lymph node metastasis, so a Transwell assay was performed on ESCC cells to assess the effect of LOXL2 silencing and overexpression on the apoptotic, migratory, and invasive capacity of ESCC cells. In TE-1 cells, the percentage of apoptosis was significantly higher after LOXL2 silencing than in the control group (*P* < 0.001) (Fig. 3a), whereas in KYSE30 cells, no significant change in the percentage of apoptosis was observed after LOXL2 overexpression (*P* = 0.679) (Fig. 3b). The above results indicated that silencing LOXL2 significantly accelerated the apoptosis of ESCC cells. To further explore the effect of LOXL2 on the migration and invasion of ESCC cells, we performed Transwell assays, and in TE-1 cells, cell migration and invasion were significantly reduced after LOXL2 silencing (migration, *P* < 0.01; invasion, *P* < 0.01) (Fig. 3c, e). In KYSE30 cells, cell migration and invasion were significantly enhanced after LOXL2 overexpression (migration, *P* < 0.05, invasion, *P* < 0.01) (Fig. 3d, f). All these results suggest that LOXL2 is involved in the regulation of apoptosis, migration and invasion in ESCC cells.

### Screening and analysis of differentially expressed genes in esophageal squamous carcinoma cells with different LOXL2 expression levels

**Fig. 4:**
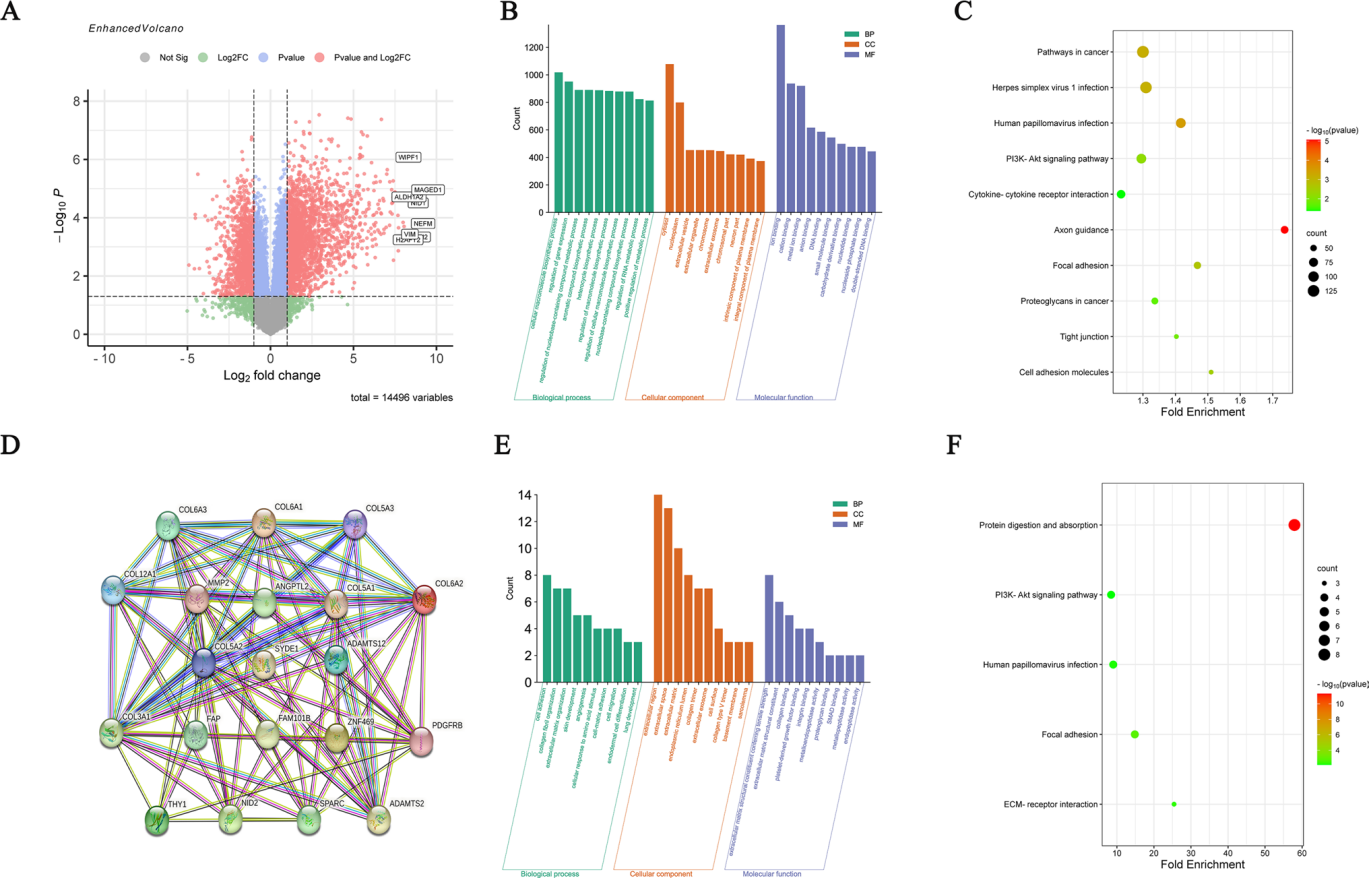
Screening and analysis of differentially expressed genes in esophageal squamous carcinoma cells with different LOXL2 expression levels. **a** DEGs were selected with a fold change > 2 and *P* value < 0.05 among the high-throughput sequencing dataset. The datasets showed an overlap of 3713 genes. **b** Analysis of GO annotations of DEGs with respect to three domains: biological process, cellular component, and molecular function. **c** KEGG pathway analysis of DEGs. The size of a dot represents the number of genes enriched for the KEGG pathway; colors from red to green represent the adjusted P value. **d** Applying the STRING database to construct co-expression networks of similar genes. **e** Analysis of GO annotations of similar genes with respect to three domains: biological process, cellular component, and molecular function. **f** KEGG pathway analysis of similar genes. The size of a dot represents the number of genes enriched for the KEGG pathway; colors from red to green represent the adjusted *P* value

In this study, LOXL2 mRNA levels were measured in six esophageal squamous carcinoma cell lines, and the highest and lowest expression levels were found in TE-1 cells and KYSE30 cells, respectively. A total of 3713 differentially expressed genes (DEGs) with the same trend were screened by high-throughput sequencing in these two cell lines, including 2527 upregulated genes and 1186 downregulated genes (Fig. 4a). The DAVID database was enriched for GO function and KEGG pathway analysis of differentially expressed genes. GO functional enrichment analysis showed that differentially expressed genes in esophageal squamous carcinoma were mainly involved in cellular macromolecule biosynthetic process, regulation of gene expression, regulation of nucleobase-containing compound metabolic process, aromatic compound biosynthetic process, heterocycle biosynthetic process and other biological processes. Molecular functions included ion binding, cation binding, metal ion binding, anion binding, DNA binding, etc. Cellular component analysis suggested that the differentially expressed genes were mainly concentrated in the cytosol and nucleoplasm (Fig. 4b). KEGG pathway enrichment analysis showed that the differentially expressed genes were mainly enriched in pathways in cancer, herpes simplex virus 1 infection, human papillomavirus infection, PI3K-Akt signaling pathway, cytokine‒cytokine-receptor interaction, etc. (Fig. 4c). Considering the serendipity of LOXL2 associated with other genes, this study used the GEPIA database to screen out genes similar to LOXL2 in esophageal cancer and applied the STRING database to construct a co-expression network (Fig. 4d) for GO and KEGG enrichment analysis of similar genes. GO functional enrichment analysis showed that similar genes in esophageal squamous carcinoma were mainly involved in collagen fibril organization, extracellular matrix organization, skin development, cell adhesion, and cellular response to amino acid stimulus. Molecular functions included extracellular matrix structural constituent conferring tensile strength, platelet-derived growth factor binding, extracellular matrix structural constituent, collagen binding, and integrin binding. Cellular component analysis suggested that similar genes were mainly concentrated in the extracellular matrix, collagen trimer, extracellular region, endoplasmic reticulum lumen, extracellular space, etc. KEGG pathway enrichment analysis showed that similar genes were mainly enriched in protein digestion and absorption, the PI3K–Akt signaling pathway, human papillomavirus infection, focal adhesion, and ECM–receptor interaction (Fig. 4f). These results suggest that LOXL2 may promote the progression of esophageal squamous carcinoma by activating the PI3K–Akt signaling pathway.

### LOXL2 regulates the PI3K/AKT signaling pathway through phosphorylation of AKT

**Fig. 5:**
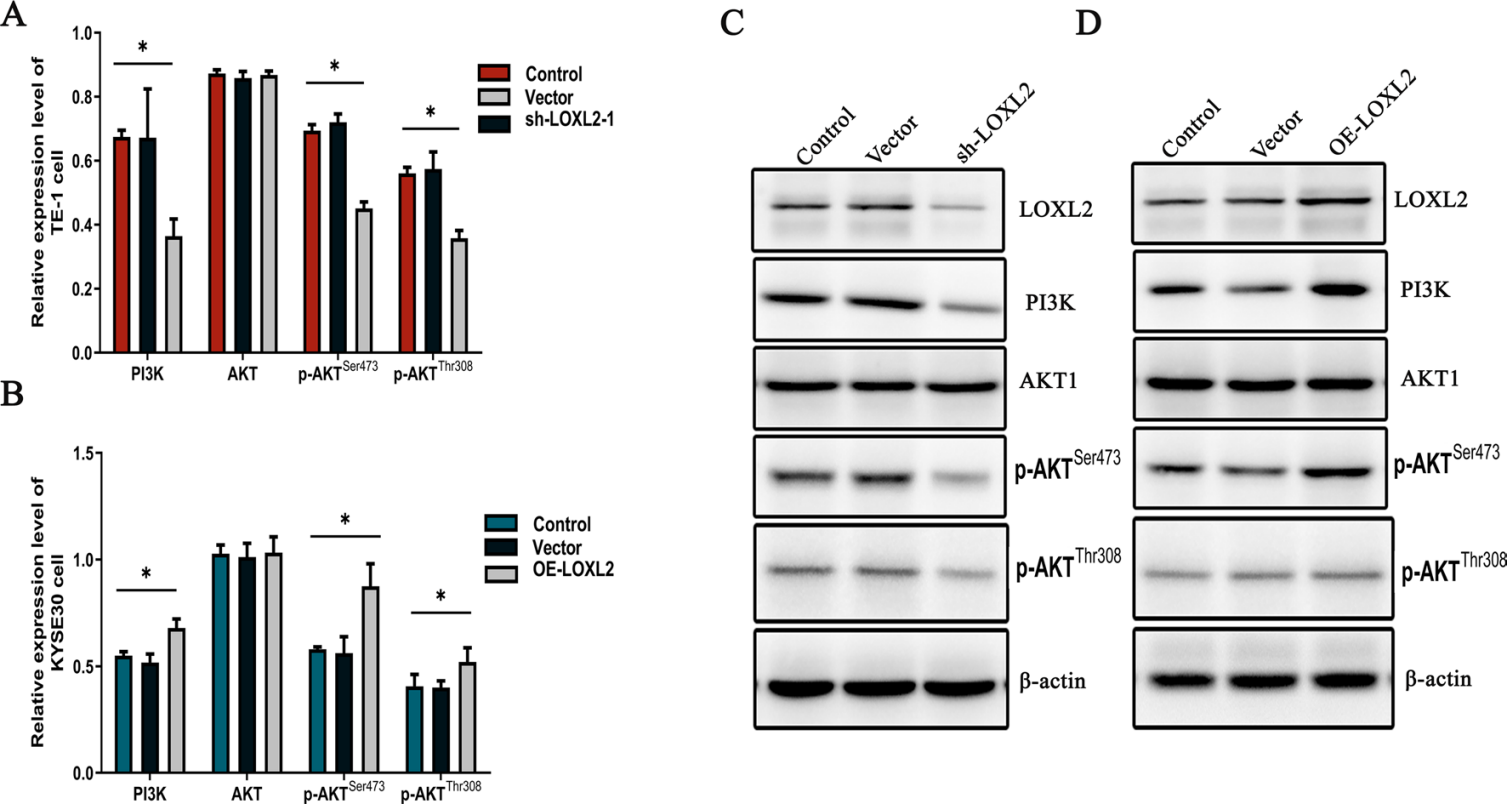
LOXL2 regulates the PI3K/AKT signaling axis through phosphorylation of AKT. **a** qRT-PCR detection of PI3K, AKT, p-AKT^Ser473^, and p-AKT^Thr308^ expression after silencing LOXL2 in TE-1 cells. **b** qRT-PCR detection of PI3K, AKT, p-AKT^Ser473^, and p-AKT^Thr308^ expression after overexpression of LOXL2 in KYSE30 cells. **c**, **d** The levels of PI3K, AKT, p-AKT^Ser473^, and p-AKT^Thr308^ proteins in TE-1 and KYSE30 cells after LOXL2 transfection were evaluated by Western blotting. P values were determined by Pearson correlation, *P < 0.05, **P < 0.01, ***P < 0.001

Bioinformatic analysis of high-throughput sequencing results suggested that LOXL2 may regulate the PI3K/AKT signaling axis to promote esophageal squamous carcinoma progression, but this still needs to be confirmed by cellular experiments. After silencing LOXL2 in TE-1 cells, the expression of PI3K, p-AKT^Ser473^ and p-AKT^Thr308^ was reduced, and no significant change in AKT expression was observed. (PI3K, *p* < 0.05, p-AKT^Ser473^, *p* < 0.001, p-AKT^Thr308^,* p* < 0.001, AKT, *p* = 0.526) (Fig. 5a). Overexpression of LOXL2 in KYSE30 cells resulted in elevated expression of PI3K, p-AKT^Ser473^, and p-AKT^Thr308^, and no significant change in AKT expression was observed. (PI3K, *p* < 0.01, p-AKT^Ser473^, *p* < 0.01, p-AKT^Thr308^, *p* < 0.05, AKT, *p* = 0.916) (Fig. 5b). Protein blotting showed that silencing LOXL2 in TE-1 cells decreased the phosphorylation levels of PI3K and AKT (Fig. 5c), while overexpression of LOXL2 in KYSE30 cells increased the phosphorylation levels of PI3K and AKT (Fig. 5d). These results suggest that LOXL2 activates the PI3K/AKT signaling pathway and promotes the proliferation, invasion and migration of esophageal squamous carcinoma through phosphorylation of AKT^Ser473^ and AKT^Thr308^.

## Discussion

The histological types of esophageal cancer (EC) are esophageal squamous cell carcinoma (ESCC) and esophageal adenocarcinoma (EAC) [31]. The main histological subtype of esophageal cancer in China is esophageal squamous carcinoma [32], which has a high incidence and shows significant geographical differences among regions in China, with Xinjiang being one of the major incidence areas [33]. Early symptoms of esophageal squamous carcinoma are often not obvious, and patients with locally advanced disease are prone to tumor invasion and metastasis [34]. In this study, the value of LOXL2 in esophageal squamous carcinoma was comprehensively investigated by analyzing the clinicopathological data of patients with locally advanced disease in Xinjiang.

LOXL2, a member of the LOX gene family, induces cross-linking of extracellular matrix collagen and elastin, remodeling the extracellular matrix and making tumors more susceptible to local infiltration and distant metastasis [10]. The results of this study showed that LOXL2 expression levels were significantly higher in esophageal squamous carcinoma tissues than in paraneoplastic tissues. In patients with locally advanced disease, positive LOXL2 expression was highly correlated with tumor T stage and lymph node metastasis, suggesting that LOXL2 may promote the progression of ESCC through its involvement in tumor proliferation and metastasis. Wu S et al. found that LOXL2 expression was aberrantly expressed in liver malignancies and could enhance the infiltration and invasion of hepatocellular carcinoma cells and promote hepatocellular carcinoma metastasis through activation of the PI3K–AKT signaling pathway [35]. Jiang L et al. found that LOXL2 could phosphorylate AKT protein expression to activate the PI3K/AKT signaling pathway and regulate the viability and apoptosis of cervical cancer cells, promoting cervical cancer progression [36]. The above findings suggest that LOXL2 plays an important role in the development of esophageal squamous carcinoma and can be an effective biomarker for determining the infiltration and metastasis of esophageal squamous carcinoma.

To further clarify the role of LOXL2 in esophageal squamous carcinoma, LOXL2 mRNA expression was measured in six groups of ESCC cells, and stable silencing and overexpression cell lines were constructed in this study. LOXL2 was silenced in TE-1 cells and overexpressed in KYSE30 cells to verify the role and molecular mechanism of LOXL2 in ESCC cell progression, and as expected, silencing and overexpression of LOXL2 greatly affected cell proliferation, migration and invasion. Notably, the apoptotic capacity of tumor cells was significantly enhanced by silencing LOXL2 but not significantly decreased by overexpression of LOXL2, but this was considered to be related to the KYSE cell background apoptotic capacity threshold. All these findings suggest that LOXL2 may have an important role in ESCC cell development.

High-throughput sequencing can sequence all DNA molecules in tissues or cells to study gene expression and function at a holistic level [37]. To further clarify the molecular mechanism of LOXL2 in the development of esophageal squamous carcinoma, in this study, high-throughput sequencing was performed on ESCC cells with relatively high and low expression of LOXL2, and the differentially expressed genes were screened using bioinformatics methods. The results of KEGG pathway enrichment analysis [38] suggested that the differentially expressed genes were highly enriched in the PI3K/AKT signaling pathway. It has been shown that the PI3K/AKT signaling pathway is activated by the lipid kinase PI3K, which aggregates to the cell membrane upon activation, phosphorylates its substrate PIP2 to IP3, binds to the PH domain-containing signaling protein AKT and phosphatidylinositol-dependent kinase (PDK), and PI3K binds to the threonine site at position 308 (Thr308) and the serine site at position 473 (Ser473) via PDK. Activated phosphorylated AKT (p-AKT) is an important mediator of this signaling pathway that translocates into the cytosol or nucleus and phosphorylates a range of substrates involved in regulating cellular functions such as survival, proliferation, differentiation and regulation of death [20]. Ediriweera M K et al. found that the PI3K/AKT signaling pathway plays an important role in the development, proliferation and progression of ovarian cancer tumors [39]. Dimri M et al. found that in hepatocellular liver cancer, activation of the PI3K/AKT pathway led to abnormalities in liver tumor cell growth, survival regulation and metabolism, which in turn led to disease progression [40].

Based on the above experimental results, to clarify the interaction between LOXL2 and the PI3K/AKT signaling pathway, this study examined the gene and protein expression levels of PI3K, AKT, p-AKT^Thr308^ and p-AKT^Ser473^ in LOXL2-silenced and LOXL2-overexpressing cell models. The results indicated that the expression levels of the PI3K, p-AKT^Thr308^ and p-AKT^Ser473^ genes and proteins were significantly reduced after LOXL2 silencing and increased after overexpression, while the expression levels of the AKT genes and proteins were not significantly different.

The above results suggest that LOXL2 regulates the PI3K/AKT signaling pathway through phosphorylation of AKT and ultimately promotes the progression of esophageal squamous carcinoma.

There are still some limitations in this study. We only explored the in vitro effects of LOXL2 in ESCC cells; therefore, more experiments are needed to confirm the effects of LOXL2 on ESCC in vivo. In addition, the application of LOXL2 and PI3K/AKT signaling pathway inhibitors is worth exploring in subsequent experiments.

In conclusion, our results show that LOXL2 phosphorylates AKT to activate the PI3K/AKT signaling pathway and significantly enhance ESCC cell progression, including cell growth, migration, and invasion. This study provides a clinical warning indicator for esophageal squamous carcinoma progression and a theoretical basis for precise individualized treatment and clinical translation.

## Data Availability

The dataset(s) supporting the conclusions of this article are included within the article. The original data can be obtained from the corresponding author.
